# Fatal Pulmonary Tumor Thrombotic Microangiopathy Associated With Disseminated Carcinomatosis of the Bone Marrow From Occult Gastric Cancer: An Autopsy-Confirmed Case

**DOI:** 10.7759/cureus.116263

**Published:** 2026-09-15

**Authors:** Shuka Furuki, Hiroyuki Kobayashi

**Affiliations:** 1 Division of Hematology, Department of Medicine, Jichi Medical University, Shimotsuke, JPN; 2 Division of Hematology, Nasu Red Cross Hospital, Ohtawara, JPN

**Keywords:** adenocarcinoma, dcbm, dic, gastric cancer, pttm

## Abstract

Pulmonary tumor thrombotic microangiopathy (PTTM) and disseminated carcinomatosis of the bone marrow (DCBM) are rare but life‑threatening metastatic complications of advanced malignancies, particularly gastric adenocarcinoma, and often present with hematological abnormalities such as disseminated intravascular coagulation and microangiopathic hemolytic anemia, making early recognition difficult. An 87-year-old woman was admitted with nausea and loss of appetite, and imaging revealed bilateral chronic subdural hematomas, pleural effusions, and multiple small pulmonary nodules. Laboratory tests showed anemia, thrombocytopenia, markedly elevated coagulation markers, and increased tumor markers, while bone marrow biopsy confirmed DCBM with poorly differentiated adenocarcinoma. Before the primary tumor site could be identified, the patient developed rapidly worsening hypoxia and died despite steroid pulse therapy. Autopsy revealed a type 4 gastric adenocarcinoma with extensive lymphatic invasion and pulmonary tumor microemboli with fibrocellular intimal proliferation, confirming PTTM as the cause of respiratory failure. This case illustrates the simultaneous occurrence of DCBM and PTTM due to advanced gastric cancer and underscores the importance of considering these conditions in patients with unexplained cytopenias, coagulation abnormalities, or acute respiratory decline when gastric adenocarcinoma is suspected.

## Introduction

Pulmonary tumor thrombotic microangiopathy (PTTM) is a rare but often fatal metastatic complication in which tumor microemboli lodge within the pulmonary arterioles and induce reactive fibrocellular intimal proliferation. This dual process leads to progressive and ultimately acute vascular occlusion, resulting in rapidly worsening pulmonary hypertension and right heart failure [1,2]. Disseminated carcinomatosis of the bone marrow (DCBM) represents another aggressive metastatic pattern characterized by diffuse marrow infiltration and a characteristic clinical triad of anemia, back pain, and bleeding tendency, often accompanied by disseminated intravascular coagulation (DIC) and leukoerythroblastosis [3-5]. Although each condition is individually recognized, their simultaneous occurrence is exceedingly rare. Premortem diagnosis remains challenging because these complications often deteriorate fulminantly and evade timely recognition. Here, we describe an elderly patient who initially presented with cytopenias and coagulation abnormalities due to DCBM and subsequently developed fulminant respiratory failure caused by PTTM. This case underscores the diagnostic difficulty and aggressive clinical trajectory when these two metastatic complications arise together and highlights the importance of considering both conditions in patients with suspected advanced gastric cancer.

## Case presentation

An 87-year-old woman with no significant past medical history was admitted to our hospital because of nausea and loss of appetite. On admission, she was awake and alert. Unenhanced computed tomography (CT) of the head revealed bilateral chronic subdural hematomas, which were evaluated by a neurosurgeon. Initial laboratory tests showed anemia and thrombocytopenia. Four days later, her level of consciousness deteriorated, prompting burr hole drainage for hematoma decompression. Her mental status improved after the procedure; however, her cytopenias continued to worsen throughout hospitalization. Suspected hematologic disease led to her referral to our division one month after the initial admission.

At the time of referral, she remained awake and alert. Her vital signs were blood pressure 113/56 mmHg, pulse rate 78 beats/minute (regular), temperature 37.7°C, and oxygen saturation 95% on 2 L/minute nasal cannula. Physical examination revealed inspiratory rales bilaterally and decreased breath sounds over the lower lung fields. No lymphadenopathy or peripheral edema was noted. The abdomen was soft and non-tender with normal bowel sounds. Scattered papules were present on the extremities. Neurological examination was unremarkable.

Laboratory evaluation demonstrated a white blood cell count of 3.9 × 10⁹/L (neutrophils 81%), hemoglobin 6.1 g/dL, and platelet count 33 × 10⁹/L. Coagulation studies showed markedly elevated D-dimer (59.8 µg/mL) and fibrin degradation products (320.0 µg/mL), with a fibrinogen level of 296 mg/dL. Blood chemistry revealed elevated C-reactive protein (11.2 mg/dL), lactate dehydrogenase (728 IU/L), alkaline phosphatase (883 IU/L), and ferritin (1895 ng/mL). Based on these parameters, the International Society on Thrombosis and Haemostasis (ISTH) DIC score was calculated as 7 (platelets 2 points, prothrombin time prolongation 2 points, markedly elevated fibrin degradation products/D-dimer 3 points, fibrinogen 0 points), fulfilling the criteria for overt DIC. However, schistocyte percentage and haptoglobin levels were not obtained at admission, limiting the ability to quantitatively evaluate microangiopathic hemolytic anemia. Tumor markers were also elevated, including carcinoembryonic antigen (59.2 ng/mL; reference: 0.1-5.0 ng/mL) and soluble interleukin-2 receptor (1,380 U/mL; reference: 188-423 U/mL) (Table 1).

**Table 1:** Laboratory findings on admission.

Parameter	Result	Reference range
Hemogram		
White blood cell count	3.9 × 10⁹/L	4.0–10.0 × 10⁹/L
Neutrophils	81%	40–70%
Lymphocytes	10%	20–40%
Monocytes	5%	2–8%
Basophils	1%	0–1%
Eosinophils	1%	1–6%
Myelocytes	2%	0%
Red blood cell count	1.98 × 10¹²/L	4.3–5.7 × 10¹²/L
Hemoglobin	6.1 g/dL	13.5–17.5 g/dL
Mean corpuscular volume	85.9 fL	80–100 fL
Mean corpuscular hemoglobin	30.8 pg	27–34 pg
Mean corpuscular hemoglobin concentration	35.9 g/dL	32–36 g/dL
Platelet count	33 × 10⁹/L	150–400 × 10⁹/L
Reticulocytes	25‰	5–15‰
Biochemistry		
C-reactive protein	11.2 mg/dL	<0.3 mg/dL
Albumin	2.6 g/dL	3.8–5.3 g/dL
Total bilirubin	1.0 mg/dL	0.3–1.2 mg/dL
Aspartate aminotransferase	60 U/L	10–40 U/L
Alanine aminotransferase	13 U/L	5–45 U/L
Alkaline phosphatase	883 U/L	115–359 U/L
Gamma-glutamyl transferase	50 U/L	10–70 U/L
Lactate dehydrogenase	728 U/L	120–240 U/L
Blood urea nitrogen	21.6 mg/dL	8–20 mg/dL
Creatinine	0.58 mg/dL	0.65–1.07 mg/dL
Sodium	127 mEq/L	138–145 mEq/L
Potassium	3.4 mEq/L	3.6–4.8 mEq/L
Chloride	98 mEq/L	101–108 mEq/L
Calcium	7.9 mg/dL	8.8–10.1 mg/dL
Blood glucose	131 mg/dL	70–109 mg/dL
Serum iron	114 µg/dL	54–181 µg/dL
Unsaturated iron-binding capacity	48 µg/dL	111–255 µg/dL
Ferritin	1,895 ng/mL	30–400 ng/mL
Coagulation		
Prothrombin time	20.4 seconds	10–13 seconds
Fibrin degradation products	320.0 µg/mL	<5.0 µg/mL
D-dimer	59.8 µg/mL	<1.0 µg/mL
Fibrinogen	296 mg/dL	200–400 mg/dL
Tumor markers		
Carcinoembryonic antigen	59.2 ng/mL	<5.0 ng/mL
Carbohydrate antigen 19-9	2.06 U/mL	<37 U/mL
Squamous cell carcinoma antigen	2.1 ng/mL	<1.5 ng/mL
Cancer antigen 15-3	14.9 U/mL	<25 U/mL
Cancer antigen 125	50.6 U/mL	<35 U/mL
Alpha-fetoprotein	3.3 ng/mL	<10 ng/mL
Soluble interleukin-2 receptor (sIL-2R)	1,380 U/mL	122–496 U/mL

Unenhanced CT of the chest and abdomen showed bilateral pleural effusions and multiple small pulmonary nodules (Figure 1A). No lymphadenopathy, hepatosplenomegaly, or infectious foci were identified. Bone marrow biopsy revealed clusters of poorly differentiated adenocarcinoma infiltrating the marrow (Figure 1B). Based on the clinical presentation, laboratory abnormalities, imaging findings, and histopathology, a diagnosis of DCBM due to adenocarcinoma was made. The bleeding tendency associated with DCBM was considered a possible contributor to her bilateral chronic subdural hematomas.

**Figure 1:**
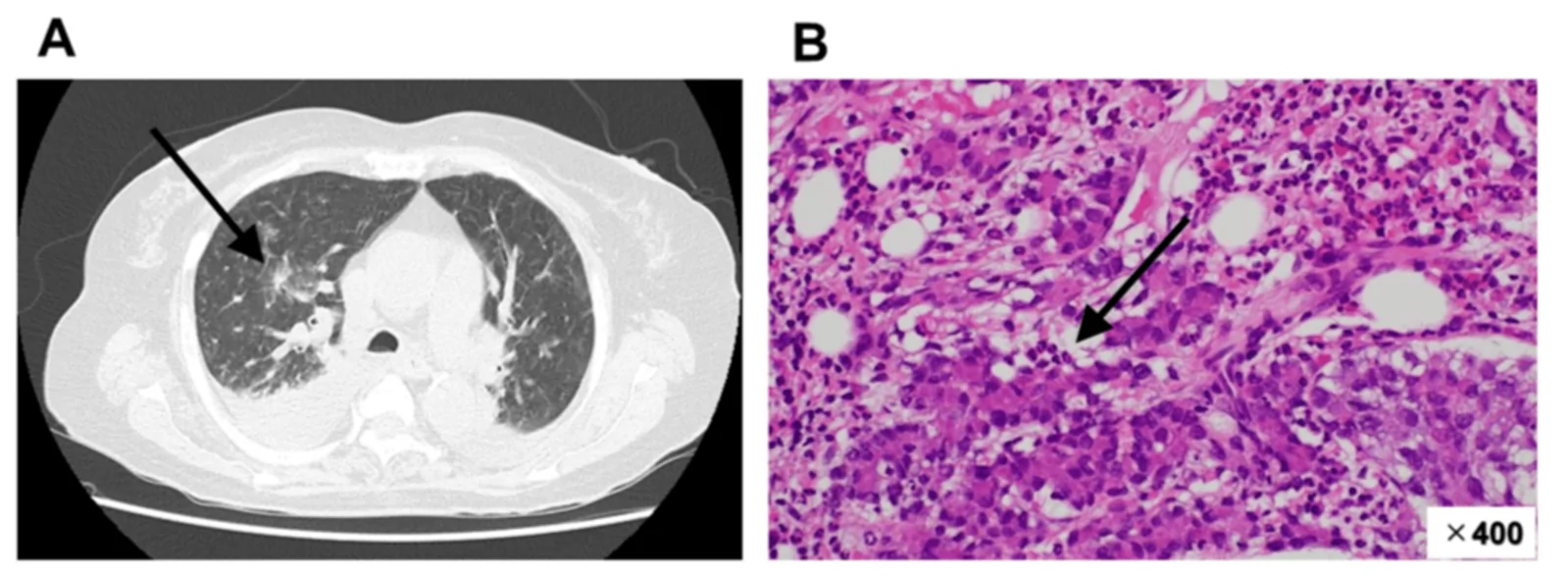
Chest computed tomography and bone marrow findings at diagnosis. (A) Unenhanced chest computed tomography showing multiple centrilobular nodules (arrow) in both lungs and bilateral pleural effusions. No hilar or mediastinal lymphadenopathy was identified. (B) Bone marrow biopsy demonstrating diffuse proliferation of poorly differentiated adenocarcinoma (arrow) (hematoxylin and eosin staining).

Endoscopic evaluation of the gastrointestinal tract and bronchi was planned to identify the primary tumor. However, on the third day after referral, she developed rapidly worsening hypoxia, necessitating cancellation of the procedure. High-dose methylprednisolone pulse therapy was initiated, but her respiratory status continued to decline, and she died the following day.

An autopsy was performed to determine the primary origin of the adenocarcinoma and the cause of her rapid respiratory failure. Gross examination revealed a type 4 gastric tumor measuring 60 × 35 mm with hemorrhage located on the lower body and anterior wall (Figure 2A). Histological analysis showed poorly differentiated, non-solid adenocarcinoma with extensive lymphatic invasion (Figures 2B, 2C). Microscopic examination also identified distant metastases in the spleen and lymph nodes. The lungs demonstrated widespread tumor microemboli within the pulmonary arterioles, accompanied by fibrocellular intimal proliferation, organized thrombi, and recanalization (Figures 3A-3D). These lesions were diffusely distributed across bilateral lobes, with a substantial proportion of small pulmonary arterioles involved. Although precise quantification was not feasible, the extent of arteriolar involvement was sufficient to cause critical obstruction of the pulmonary microvasculature. These findings confirmed metastatic gastric cancer causing both DCBM and PTTM, ultimately leading to respiratory failure and death.

**Figure 2:**
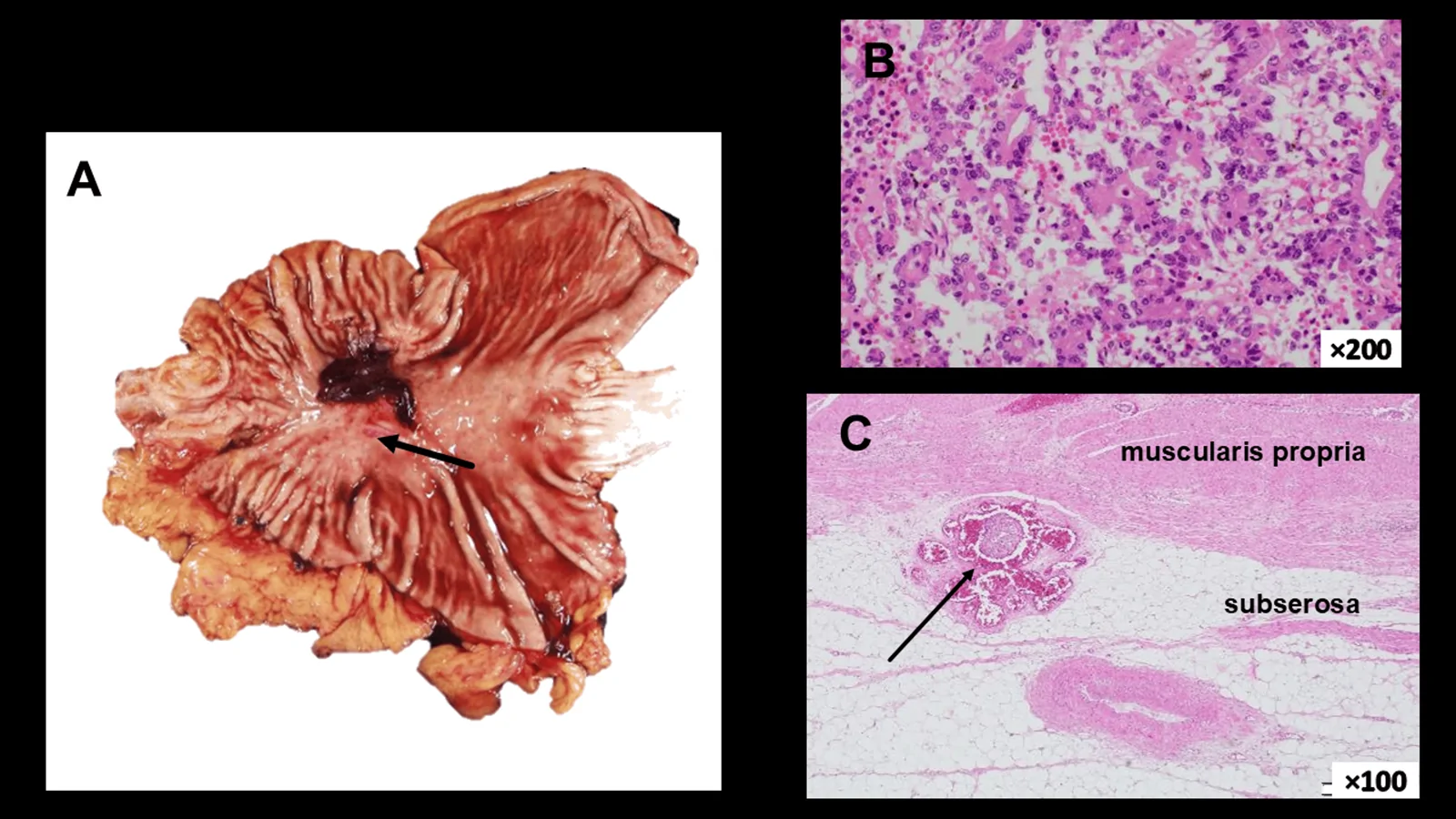
Autopsy findings of the stomach. (A) Gross examination revealing a type 4 gastric tumor measuring 60 × 35 mm with hemorrhage, located on the lower body and anterior wall of the stomach (arrrow). (B) Microscopic section showing diffuse proliferation of poorly differentiated, non‑solid adenocarcinoma (hematoxylin and eosin staining, ×200). (C) Extensive lymphatic invasion by tumor cells within the subserosal layer.

**Figure 3:**
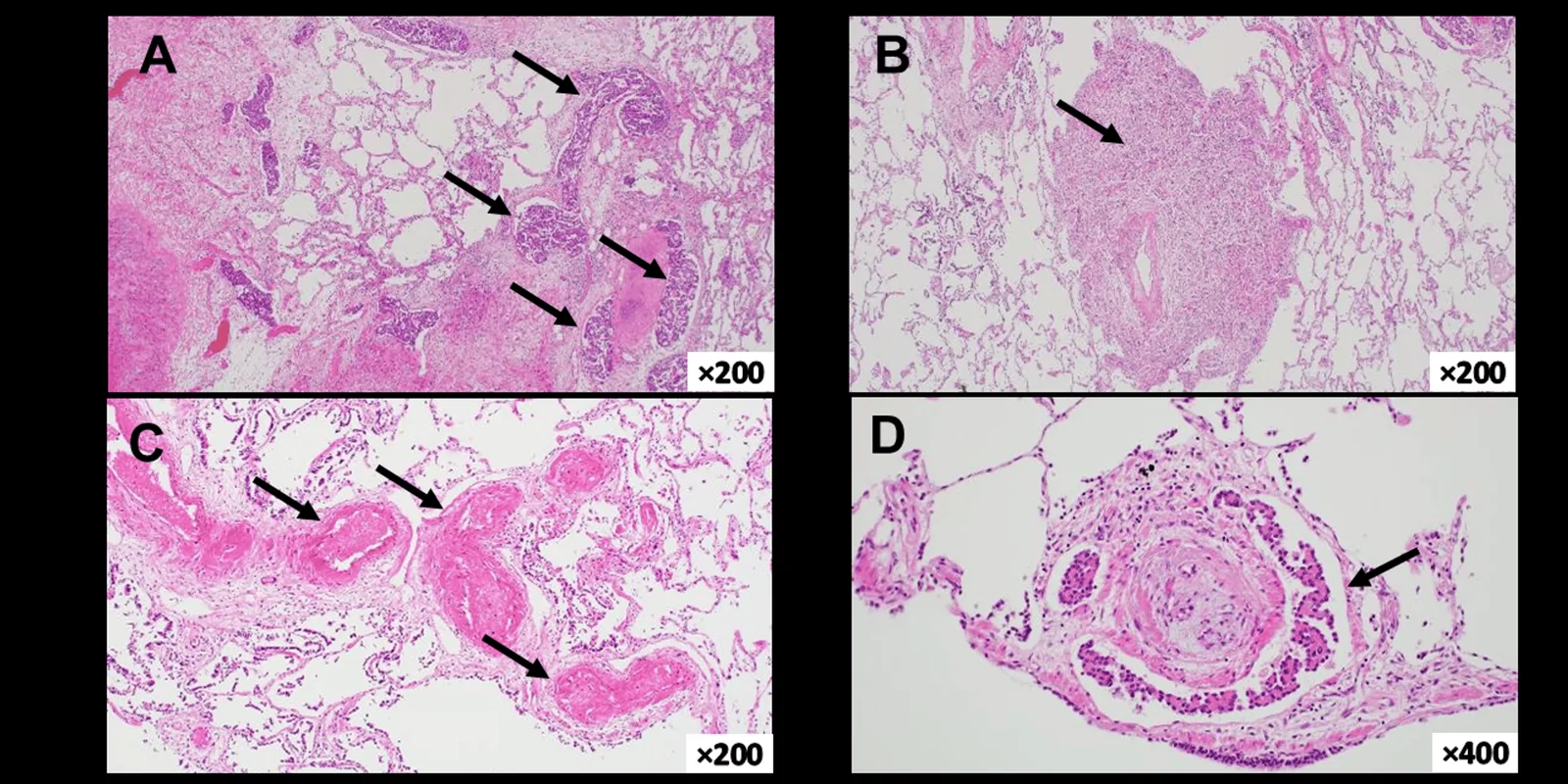
Autopsy findings of the lung (hematoxylin and eosin staining). (A) Pulmonary arterioles containing tumor microemboli (arrow) with marked luminal obstruction (×200). (B) Fibrocellular intimal proliferation (arrow) causing concentric narrowing of the arterial lumen (×200). (C) Organized thrombus (arrow) within a small pulmonary artery, accompanied by marked mural thickening (×200). (D) High‑magnification view showing recanalization channels (arrow) within an organized thrombus (×400).

## Discussion

We encountered a case involving the concomitant occurrence of DCBM and PTTM due to advanced gastric cancer. The patient died before the primary tumor site could be identified and before PTTM was recognized as the cause of respiratory failure. A retrospective review of the initial CT scan revealed multiple small pulmonary nodules, which likely represented early manifestations of PTTM. The bilateral pleural effusions observed were also consistent with right heart failure secondary to PTTM. A contrast-enhanced CT angiogram was not performed because the patient developed rapidly worsening respiratory status shortly after referral, which prevented further imaging studies.

DCBM results from widespread metastasis of solid tumors to the bone marrow [3,4]. It is frequently accompanied by hematologic abnormalities such as DIC, leukoerythroblastosis, and microangiopathic hemolytic anemia (MAHA) [3-7]. In contrast, PTTM is an uncommon but severe metastatic complication characterized by tumor microemboli within the pulmonary arterioles, fibrocellular intimal proliferation, and organized thrombus formation. These pathological changes lead to increased pulmonary vascular resistance, rapidly progressive pulmonary hypertension, and acute right heart failure [1,2]. Radiological findings such as ground-glass opacities, small nodules, and tree‑in‑bud patterns have been reported as characteristic features of PTTM [2,8,9]. However, diagnosing PTTM premortem is extremely challenging due to its rarity, non-specific imaging findings, and fulminant clinical course [1,2,9]. In our case, the coexistence of PTTM was not suspected at the time DCBM was diagnosed, and the patient’s rapid deterioration left no opportunity for further evaluation.

Both DCBM and PTTM represent aggressive patterns of distant metastasis. Patients with either condition often exhibit multiple metastatic sites, making their coexistence unsurprising. Uruga et al. reported that among 30 autopsy-confirmed PTTM cases, nine demonstrated bone marrow infiltration [10]. However, to our knowledge, no prior publication has precisely described a case in which DCBM and PTTM were confirmed to occur simultaneously. This case therefore represents the first detailed report of PTTM occurring alongside DCBM.

Interestingly, DCBM and PTTM share several clinical and pathological features. First, gastric cancer, particularly poorly differentiated adenocarcinoma and signet‑ring cell carcinoma, is the most frequent primary malignancy associated with both conditions [1-5,7,11,12]. Second, both entities are commonly accompanied by hematologic dysfunctions such as DIC and MAHA [2-7,11,13]. In our case, the patient had poorly differentiated gastric adenocarcinoma confirmed histopathologically and exhibited DIC at the time of initial admission. The underlying reason for these shared features remains unclear, but the overlap suggests that clinicians should consider both DCBM and PTTM when encountering advanced gastric cancer with hematologic abnormalities such as DIC or MAHA.

## Conclusions

This case illustrates that PTTM and DCBM can occur simultaneously in patients with advanced gastric cancer. Hematologic abnormalities, particularly DIC and MAHA, may provide important early clues to these rapidly progressive metastatic complications. Because both PTTM and DCBM often present with non-specific findings and deteriorate quickly, early recognition is essential. Clinicians should consider these entities when encountering unexplained cytopenias, coagulation abnormalities, or acute respiratory decline in patients with advanced malignancy. Increased awareness of the potential coexistence of PTTM and DCBM may support earlier diagnostic consideration and improve clinical understanding of these rare but fatal manifestations of advanced cancer.
